# Comparison of Neoadjuvant Chemotherapy Efficiency in Advanced Ovarian Cancer Patients Treated With Paclitaxel Plus Carboplatin and Intraperitoneal Bevacizumab vs. Paclitaxel With Carboplatin

**DOI:** 10.3389/fmed.2022.807377

**Published:** 2022-03-09

**Authors:** Yin Tao, Xue-Ting Tang, Xing Li, An-Shan Wu, Hou-Shen Zhou, Cheng-fang Zhou

**Affiliations:** ^1^Zhuzhou Central Hospital, Zhuzhou, China; ^2^Hengyang Key Laboratory of Neurodegeneration and Cognitive Impairment, Hengyang Medical College, Institute of Neuroscience, University of South China, Hengyang, China; ^3^School of Basic Medical Sciences, Shaoyang University, Shaoyang, China; ^4^The First Affiliated Hospital of Army Medical University, Chongqing, China

**Keywords:** neoadjuvant chemotherapy, bevacizumab, intraperitoneal perfusion, advanced ovarian cancer, R0 resection

## Abstract

**Objective:**

This study evaluated the role of neoadjuvant chemotherapy (NACT) with bevacizumab intraperitoneal perfusion in advanced ovarian cancer (AOC).

**Methods:**

In this study, 80 patients with advanced epithelial ovarian cancer (stage IIIc or IV) who received NACT at the Central Hospital of Zhuzhou between February 2019 and October 2020 were enrolled. Patients were randomized to receive paclitaxel plus carboplatin (TC) or TC plus intraperitoneal perfusion of bevacizumab (TCB). The effect of chemotherapy was assessed following two cycles of chemotherapy. Cancer antigen 125 (CA125), tumor size, ascites volume, bleeding volume, duration of operation, surgical satisfaction rate, complication rate, and residual tumor were assessed to monitor response to chemotherapy.

**Results:**

Treatment with TCB regimen significantly reduced serum levels of CA125 and ascites volume (*p* < 0.001). Meanwhile, the TCB group had significantly lower intraoperative blood loss and shorter operation time (*p* < 0.001). Most importantly, patients treated with TCB regimen had a higher surgical satisfaction rate (*p* < 0.01). Moreover, the incidence of postoperative wound infection, hypoproteinemia, abdominal distension, and fever was lower in the TCB group compared with the TC group. Assessment of adverse reactions during chemotherapy showed no severe complications between the two groups.

**Conclusions:**

The results demonstrated that the TCB regimen is superior to the TC regimen alone in the treatment of AOC. These findings could help improve the surgical satisfaction rate, provide more effective treatment strategies to prolong progression-free survival and reduce postoperative complications, and promote surgical recovery in AOC.

## Introduction

**Figure 1:**
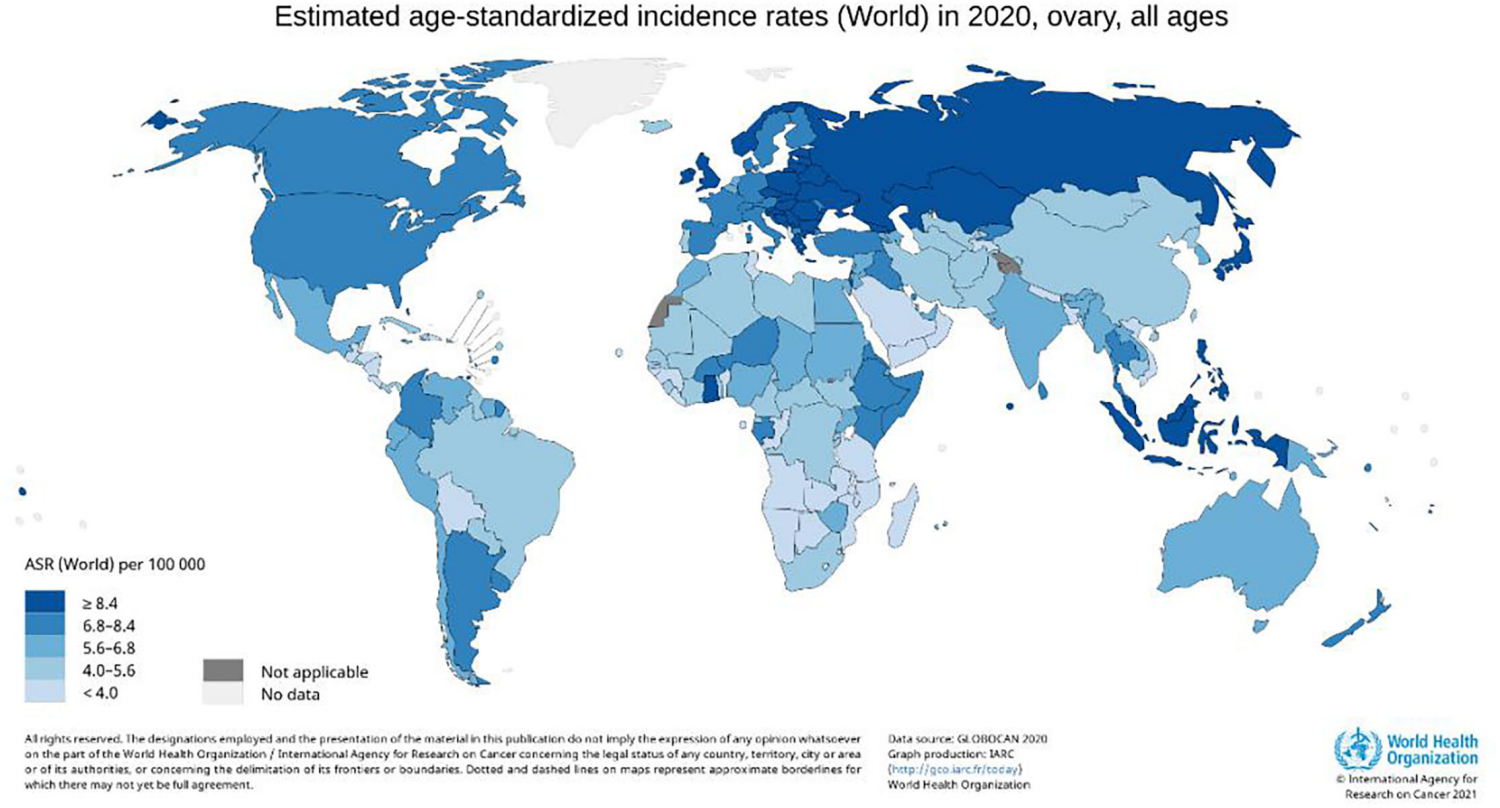
Global incidence of ovarian cancer in 2020. ASR indicates age-standardized rate.

Ovarian cancer is one of the most common malignancies of female reproductive organs after cervical and endometrial cancer (1). Epithelial ovarian cancer is the most common type of ovarian cancer (2), and the leading cause of death from gynecologic malignancies in women worldwide (3, 4) (Figure 1). About 70% of patients with ovarian cancer are advanced, and 70% relapse after surgery (5, 6). This could be because factors such as the deep-seated location of the ovaries within the pelvic cavity, small tumor volume, and lack of characteristic symptoms hinder diagnosis. Despite the increased use of chemotherapeutic drugs and maintenance treatment emphasized in the National Comprehensive Cancer Network (NCCN) guidelines, surgical debulking procedures remain the most effective treatment for ovarian cancer (7), with R0 resection repeatedly emphasized (8). However, multiple metastases to the peritoneum, mesenteric, and intestinal surfaces in patients with advanced ovarian cancer (AOC, stage III-IV) complicate surgical treatment (9). In addition, it is more challenging to achieve R0 resection in older patients with underlying diseases who cannot tolerate long-term surgery (10). To achieve R0 resection for stage III-IV AOC, several studies have proposed neoadjuvant chemotherapy (NACT), which is also recommended by NCCN (11).

Paclitaxel plus carboplatin (TC) regimen is a classical chemotherapy regimen for ovarian cancer (12). NACT followed by interval debulking surgery was superior to primary debulking surgery followed by chemotherapy as a treatment option for bulky stage IIIc or IV ovarian carcinoma (13). In addition, intravenous (i.v.) paclitaxel plus intraperitoneal (i.p.) carboplatin and paclitaxel improved survival in patients with optimally debulked stage III ovarian cancer (14). Mounting evidence has suggested that NACT can reduce postoperative complications and improve the quality of life of patients (15–17).

Bevacizumab (BV) is a recombinant human monoclonal IgG1 antibody that acts by inhibiting the biological activity of human vascular endothelial growth factors (18). BV is widely used in treating multiple tumors, including metastatic colorectal cancer, non-small cell lung cancer, metastatic breast cancer, renal cell carcinoma, glioblastoma, and other cancers, by anti-angiogenesis and changing the tumor microenvironment (19). The incorporation of i.p. chemotherapy for malignancies was first investigated in the 1980s (20). Intraperitoneal delivery of chemotherapy enhances drug delivery at the peritoneal surface and may improve outcomes by efficiently eliminating residual microscopic peritoneal disease compared with i.v. administration of chemotherapy drugs (21). Combination treatment of i.v. and i.p. chemotherapy has been shown to prolong the overall survival after primary cytoreductive surgery among patients with stage III ovarian cancer (14, 22, 23). Therefore, the efficacy of TC chemotherapy combined with an i.p. infusion of BV against AOC was explored.

In this study, 80 patients with AOC were randomly divided into two groups: TC and TC plus i.p. perfusion BV (TCB) groups. After chemotherapy, all patients underwent tumor reduction surgery. Preoperative (cancer antigen 125 (CA125) serum level, ascites volume, and tumor size), intraoperative (bleeding volume, duration of operation, and residual tumor), and postoperative (complication rate and surgical satisfaction rate) conditions of the patients were evaluated. It was found that the TCB regimen was superior to the TC regimen alone in the treatment of AOC. TCB is a promising regimen for NACT in AOC.

## Methods

### Patients

**Figure 2:**
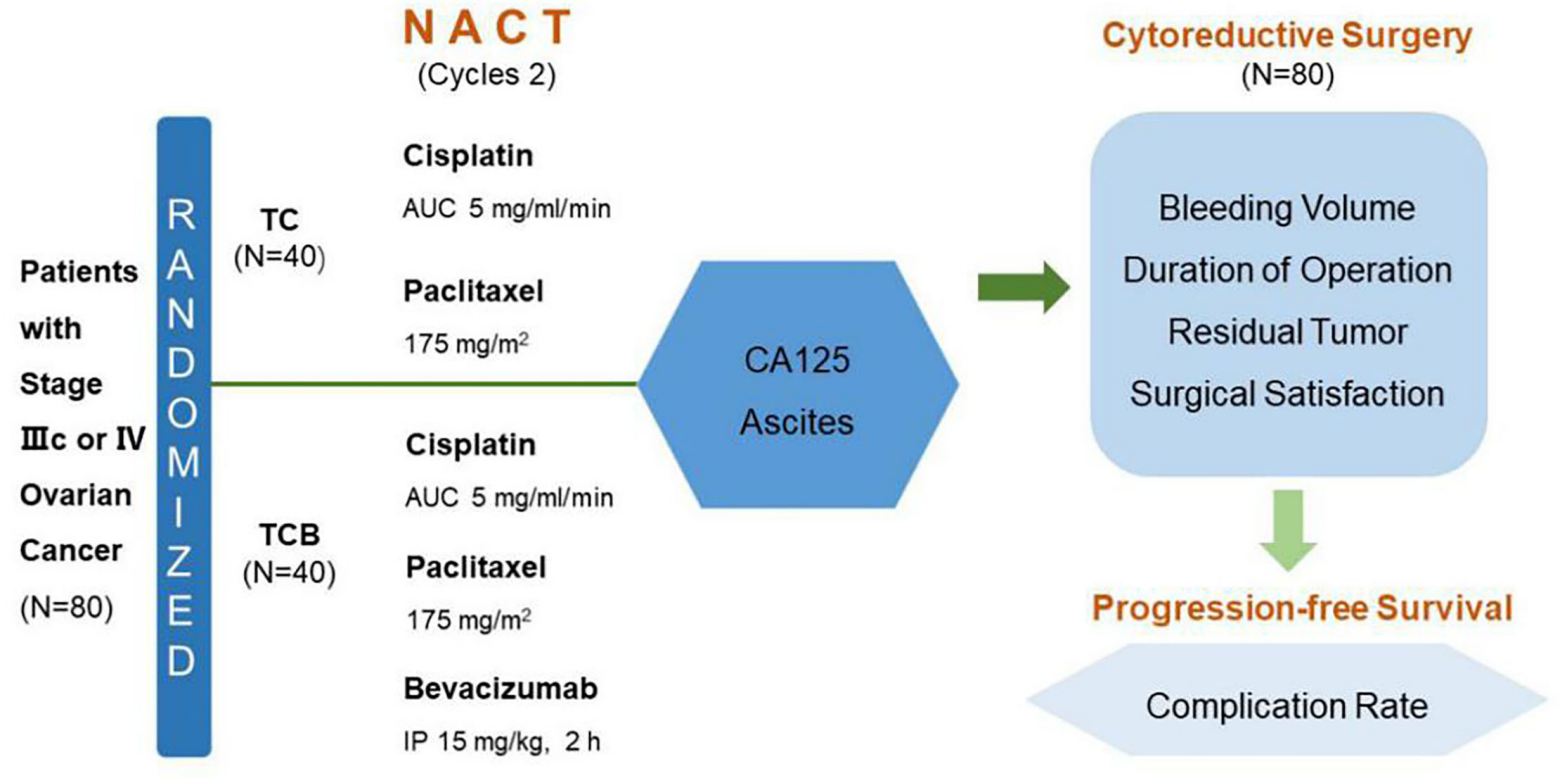
Study design. TC, carboplatin plus paclitaxel chemotherapy; TCB, carboplatin plus paclitaxel combined with bevacizumab intraperitoneal, NACT, neoadjuvant chemotherapy; AUC, area under the curve IP, intraperitoneal perfusion.

From February 2019 to October 2020, 80 cases were enrolled for NACT assessment. In total, 80 patients with histologically confirmed high-risk International Federation of Gynecology and Obstetrics (FIGO) stage IIIc/IV epithelial ovarian carcinoma received NACT from February 2019 to October 2020 at the Zhuzhou Central Hospital (Hunan, China) were enrolled. Eligibility criteria included B-ultrasound and CT diagnosis, CA125 positive, and ascites volume >1,000 ml (ultrasonic evaluation). These conditions suggested that primary surgery could not achieve adequate cytoreduction. In addition, recruited patients had no chemotherapy contraindications, a Karnofsky performance status (KPS) > 60, and life expectancy > 3 months. All patients signed the written informed consent. This study was approved by the ethics committee of Zhuzhou Central Hospital (Figure 2).

### Study Design

In total, 80 patients were randomly divided into two groups (*n* = 40): TC and TCB groups. According to the 2019 NCCN guidelines, 2–4 courses of treatment were selected for NACT in AOC. The TC group was subjected to i.v. infusion of taxol (also named as paclitaxel) at a dose of 175 mg/m^2^ of body-surface area and carboplatin at a dose equivalent to an area under the curve (AUC) of 5 on day 1 of a 21-day cycle, for two cycles. After a 2-cycle TC regimen and a recovery period of 2 weeks, debulking surgery was conducted. The TCB group was intravenously administered with 175 mg/m^2^ taxol and AUC 5 carboplatin plus i.p. perfusion of 7.5 mg/m^2^ BV (in 2,000 ml saline) over 6 h, on day 1 of a 21-day cycle, for two cycles. (Note: After the addition of BV the patients' position was changed every 2 h). For i.p. perfusion, the peritoneal drainage catheter was firstly implanted under ultrasound guidance. After 24 h of perfusion, an excessive volume of fluid in the peritoneal cavity was drained *via* the catheter. After a 2-cycle TCB regimen, patients were allowed to recover for 4 weeks and then underwent cytoreductive surgery.

### Study Assessments

Disease status assessments (physical examination, vital signs, laboratory safety assessments, and measurement of the serum CA125 level were performed at the beginning of each cycle of chemotherapy and before the initiation of cytoreductive surgery. Safety and toxicity evaluations, including chemotherapy and surgery complications (bloating [> 3 days], wound infection, hypoproteinemia [albumin <30 g/L], anemia [bemoglobin <90 g/L], fever [persisting for more than 48 h, and body temperature > 38.5°C]), were closely monitored during treatment. After completion of treatment, duration of operation, loss of blood during surgery, residual tumor size (satisfactory tumor resection was defined as tumors with residual area <1 cm in diameter, and tumors with residual area > 1 cm in diameter were defined as unsatisfactory) were collected and considered the primary indicators for evaluating therapeutic effects.

### Statistical Analysis

Counts and percentages were used to summarize qualitative variables and mean and standard deviation or median and range were used to summarize quantitative variables. Chi-square tests or exact conditional logistic regression analyses were used to determine associations between categorical variables, where appropriate. Statistical analysis was performed using R software (4.0.2, R Core Team, R Foundation for Statistical Computing, Vienna, Austria, 2020). Graphs were generated by GraphPad Prism 7 (GraphPad Software Inc., San Diego, USA).

## Results

### Clinicopathological Features

A total of 80 patients were diagnosed with AOC and treated at the Zhuzhou Central Hospital. They were randomly divided into two groups, containing 40 cases in each group. Among them, 40 subjects received a TC regimen plus bevacizumab intraperitoneal perfusion, namely a TCB regimen. The other 40 subjects were treated with a TC regimen and considered the control group. None had received hormone replacement therapy before entering the study. Between February 2019 and October 2020, 80 patients were screened for age, FIGO stage, and tumor type, without differences between the two groups at the Zhuzhou Central Hospital study sites (Table 1).

**Table 1:** Demographic and clinical features of ovarian cancer subjects.

**Variable**	**TC groups**		**TCB groups**	
	**No**.	**n (%)**	**No**.	**n (%)**
**Age**				4
≤ 55	23	57.5	18	5.0
> 55	17	42.5	22	55.0
**Tumor type**				
Serous carcinoma	25	62.5	28	70
Mucinous carcinoma	3	7.5	2	5.0
Endometrioid carcinoma	8	20.0	7	17.5
Clear cell carcinoma	4	10.0	3	7.5
**FIGO Stage**				
IIIc	33	82.5	32	80.0
IV	7	17.5	8	20

^*^represents P < 0.05. ^*^Estimates from exact conditional logistic regression analysis.

### TCB Regimen Improves Preoperative Outcomes in Patients With AOC

**Figure 3:**
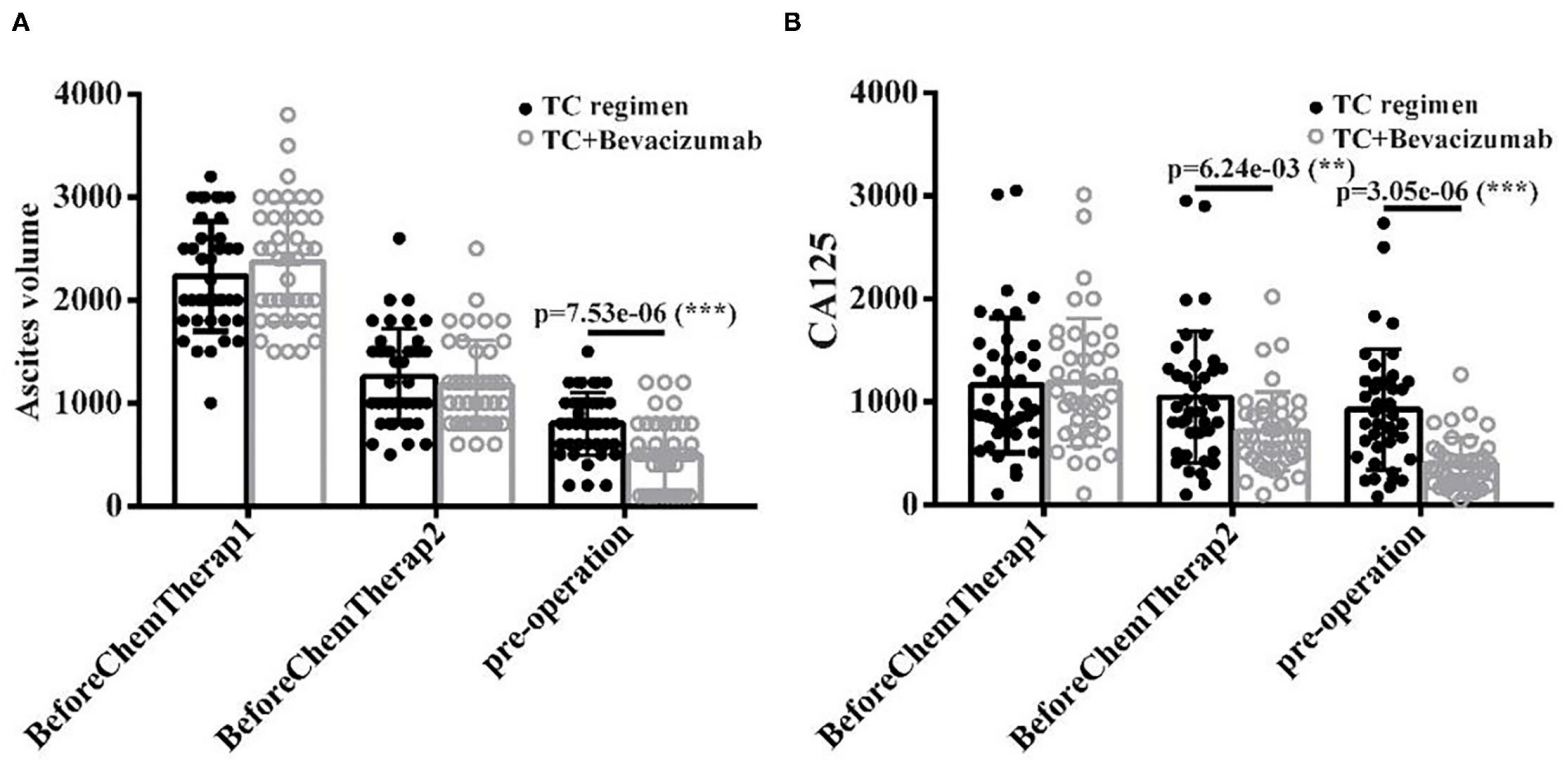
**(A,B)** The level of ascites volume and CA125 after TC and TCB treatment in advanced ovarian cancer, ** *P* < 0.01, *** *P* < 0.001, vs. control group.

Ascites volume and serum CA125 levels were measured at three different stages (Cycle1, Cycle 2, and Pre-operation). Results showed that ascites volume was significantly reduced in the TCB group than in the TC group, particularly at the pre-operation stage (after cycle 2) (*p* < 0.001, Figure 3A). Similarly, serum CA125 levels were significantly reduced in the TCB group compared with the TC group (*p* < 0.001, Figure 3B). These data indicated that the TCB regimen effectively facilitated preoperative preparations and cytoreductive surgery in patients with AOC.

### TCB Regimen Improved the Prognosis in Patients With AOC

**Figure 4:**
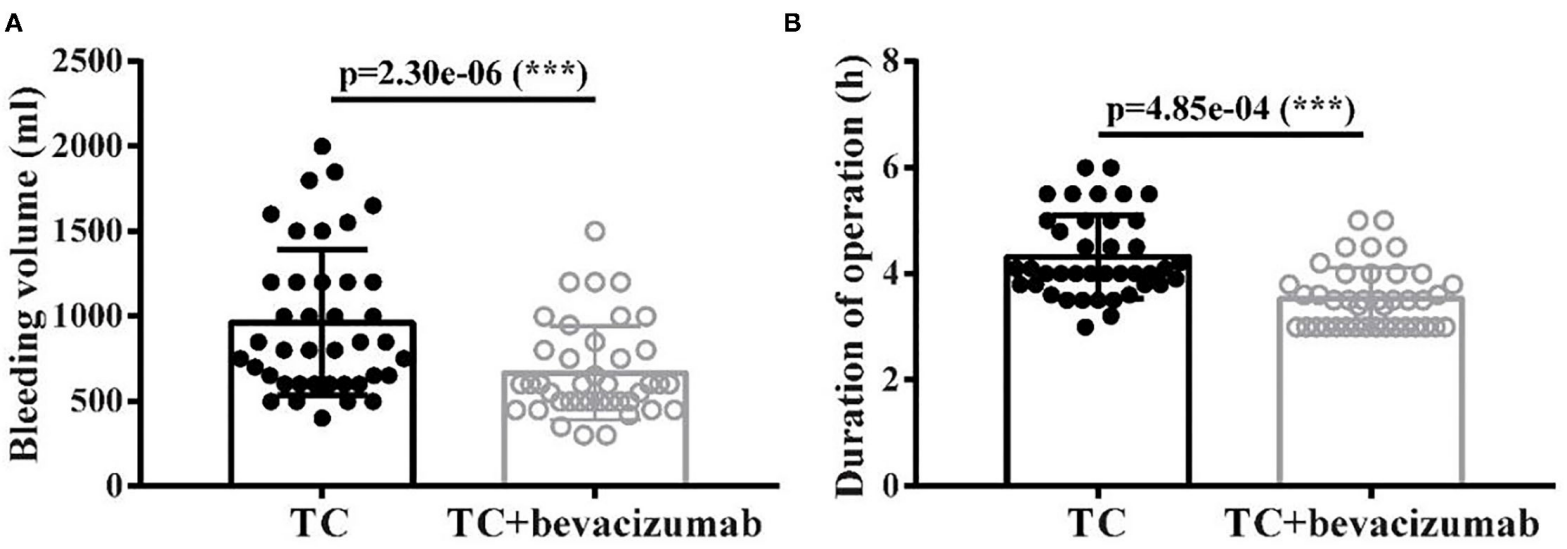
Bleeding volume **(A)** and duration of operation **(B)** after TC and TCB treatment in advanced ovarian cancer. *** *P* < 0.0001, vs. control group.

**Figure 5:**
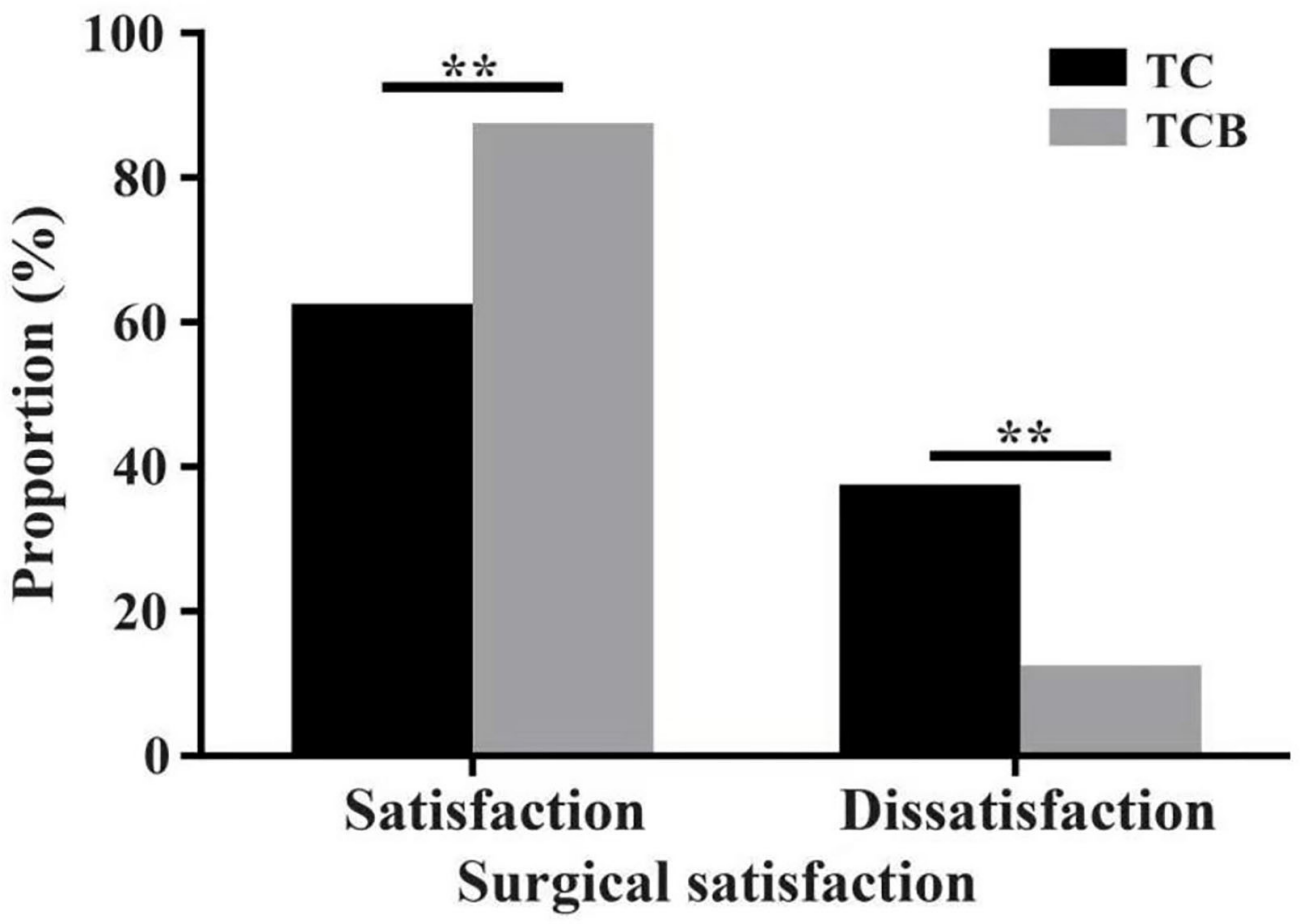
The proportion of cytoreductive surgical satisfaction after TC and TCB treatment in advanced ovarian cancer. * *P* < 0.0001, vs. control group. **represents *P* < 0.01.

The size of residual disease after cytoreduction is one of the most crucial prognostic factors for patients with ovarian cancer (24). After undergoing two cycles of TC and TCB regimen, respectively, cytoreductive surgery was performed on all 80 patients. We found that intraoperative bleeding volume (*p* < 0.001, Figure 4A) was reduced, and operative duration (*p* < 0.001, Figure 4B) was significantly shorted in the TCB group during the operation. In this study, minimal residual disease (<1 cm) was achieved in 25/4the 0 of the TC group but increased to 33/40 in the TCB group. Gross residual disease (>1 cm) was 15/40 but primarily decreased to 7/40 in the TCB group (*p* < 0.01; Table 2, Figure 5).

**Table 2:** Diameter of residual tumor size in two groups.

**Residual tumor size**	**Group**	**No. of patients**
<1 cm	TC	25
	TCB	33
1–2 cm	TC	11
	TCB	4
>2 cm	TC	4
	TCB	3

These data demonstrated that the TCB regimen improved the prognosis of patients with AOC.

### TCB Regimen Reduces Postoperative Complications and Promotes Surgical Recovery in Patients With AOC

To assess the effect of the TCB regimen, the complication of the patients was recorded post cytoreductive surgery. Abdominal distension and hypoproteinemia were the common complication type comprising 13 cases (32.5%) of the TC group patients, whereas the TCB group was found in nine patients (22.5%) (95% CI = 1.675, 1.525–1.827). In the TCB group, the incidence of pyrexia was only 5% (two cases), while in the TC group was 12.5% (five cases) (95% CI = 1.913, 1.849–1.976). Simultaneously, postoperative wound infection accounted for only 2.5% (one case) in the TCB group, but the incidence was 7.5% (three cases) in the TC group (95% CI = 1.95, 1.901–1.999). Therefore, we can conclude that the TCB regimen prominently lessened the quality of life of patients who performed cytoreductive surgery by reducing the rate of postoperative complication (Table 3).

**Table 3:** Cytoreductive surgery of ovarian cancer subjects.

**Variable**	**TC groups**		**TCB groups**		**OR**
	**No**.	**n (%)**	**No**.	**n (%)**	(95% CI)^*^
**Complication**					
Abdominal distension	13	32.5	9	22.5	1.675 (1.523–1.827)
Pyrexia	5	12.5	2	5.0	1.913 (1.849–1.976)
Hypoproteinemia	13	32.5	9	22.5	1.725 (1.625–1.825)
Wound infection	3	7.5	1	2.5	1.950 (1.901–1.999)

* *Estimates from exact conditional logistic regression analysis*.

Given the chemotherapy side effects of bevacizumab, we also detected chemotherapy complications. The gastrointestinal reaction is the most common chemotherapy complication of bevacizumab. We demonstrated that the incidence of gastrointestinal reaction has no significant difference between TC and TCB groups (95% CI = 1.675, 1.57–1.78). Granulocytopenia (95% CI = 1.813, 1.725-1.9), thrombocytopenia (95% CI = 1.825, 1.74–1.91), and fever (95% CI = 1.938, 1.883–1.992), as well as liver dysfunction (95% CI = 1.925, 1.866-1.984), among others, showed no evident change both in TC and TCB groups (Table 4). No patients in the TCB group discontinued bevacizumab because of severe side effects, which further indicated that TCB therapy produces better results than TC therapy in patients with AOC without serious side effects.

**Table 4:** Chemotherapy complications of ovarian cancer subjects.

**Complications**	**TC groups**		**TCB groups**		**OR**
	**No**.	**N (%)**	**No**.	**n (%)**	(95% CI)^*^
Gastrointestinal reaction	12	62.5	14	82.5	1.675 (1.570–1.780)
Granulocytopenia	8	37.5	7	17.5	1.813 (1.725–1.900)
Trombocytopenia	6	27.5	8	10.0	1.825 (1.740–1.910)
Fever	2	5.0	3	7.5	1.938 (1.883–1.992)
Liver dysfunction	4	10.0	2	5.0	1.925 (1.866–1.984)
Nervous system	3	7.5	1	2.5	1.950 (1.901–1.999)
Renal dysfunction	0	0	0	0	
hypertension	0	0	2	5.0	
Bowel perforation	0	0	0	0	

* *Estimates from exact conditional logistic regression analysis*.

## Discussion

R0 resection is an essential factor in the prognosis of AOC. In this study, the TCB chemotherapy regimen was used to significantly improve the satisfaction of surgical tumor reduction in patients with AOC. Some clinical trials of AOC demonstrated that complete removal of tumor residual disease (RD, 0 cm) can achieve a better prognosis and prolong progression-free survival (PFS) (25–28). A retrospective study of AOC (FIGO stage III) showed that the overall survival was 71.9, 42.4, and 35 months for patients with RD of 0 cm, <1 cm, and >1 cm, respectively (13), suggesting that overall survival is not dependent on the RD. In a randomized phase II trial by the European Organization for Research and Treatment of Cancer (EORTC) and National Cancer Institute of Canada (NCIC) (29), 632 patients with AOC (FIGO stage III/IV) were randomly assigned to NACT (paclitaxel and carboplatin, TC regimen) or primary debulking surgery (PDS) group. It was found that 80.6% of patients in the NACT group had RD >1 cm compared with 41.6% in the PDS group. These clinical trials confirmed that NACT in patients with AOC could improve R0 resection and prolong PFS.

In our study, 80 patients with advanced-stage epithelial ovarian carcinoma were randomly assigned to receive TC or TCB regimen. It was found that compared with TC regimen, treatment with TCB regimen significantly reduced serum levels of CA125 and ascites volume. Although the level of CA125 cannot be used to assess the severity of ovarian cancer, it is an important factor in the evaluation of treatment outcomes. Specifically, a reduction in CA125 level to <50% of pre-treatment value after two cycles of platinum-based chemotherapy is associated with improved survival (30). Although their study had a small sample size, Riedinger et al. (31) in a small sample size study in advanced ovarian cancer, NACT used to ccompletely removed macroscopic disease which significantly improved the prognosis of patients with preoperative CA-125 ≤ 20 U/ml. The interval between CA125 elevation and secondary surgery is crucial for successful treatment.

Most importantly, the satisfaction of surgical tumor reduction was improved in the TCB group in our study. However, data on overall survival were lacking due to several factors, which limited our study. Petrillo et al. (32), enrolled 75 patients with unresectable high-grade serous AOC receiving NACT with or without BV. Twenty-five patients in the treatment group received the NACT regimen (TC combined with i.v. injection of BV 15 mg/m^2^) and 50 patients in the control group received i.v. injection of TC alone. About 80% of patients achieve R0 resection in the treatment group compared with 72.3% in the control group. PFS was significantly prolonged in the treatment group compared with the control group (18 vs. 10 months). In addition, no significant differences in symptoms were found between the two groups. The findings from this study are consistent with our results. Data from GOG218 (33) and ICON7 (34) trials demonstrated that TC combined with BV maintenance therapy also improved the survival time of AOC patients who could not undergo PDS.

The incorporation of i.p. chemotherapy for malignancies was first investigated in the 1980s (20). The advantages of i.p. chemotherapy include the ability of chemotherapy drugs to penetrate the peritoneal barrier, act directly on the tumor, increase the dose of chemotherapy drugs (5–30-fold higher), and reduce the toxicity of systemic chemotherapy drugs (35, 36). Therefore, 7.5 mg/kg BV was selected for i.p. perfusion. Compared with the TC regimen, the TCB regimen had a better therapeutic effect, did not increase the toxicity of chemotherapy, and improved the quality of life of patients. Moreover, the incidence of postoperative wound infection, hypoproteinemia, abdominal distension, and fever was lower in the TCB group compared with the TC group. Furthermore, the assessment of adverse reactions during chemotherapy showed no severe complications between the two groups.

A major limitation of this study is the small sample size. Therefore, further studies should be conducted with larger sample sizes. Future studies should also evaluate recurrence time and prognosis, which are not captured in the current study.

In summary, our findings demonstrated that the TCB regimen is superior to the TC regimen alone in the treatment of AOC. These findings could help improve the surgical satisfaction rate, provide more effective treatment strategies to prolong progression-free survival and reduce postoperative complications, and promote surgical recovery in AOC.

## Data Availability Statement

The raw data supporting the conclusions of this article will be made available by the authors, without undue reservation.

## Ethics Statement

This case report was approved by the Ethics Committee of Zhuzhou Central Hospital (Ethics Number 2020s1012) and approved for publication. All patients signed the written informed consent.

## Author Contributions

C-fZ participated in the design, performed statistical analyses, and drafted the manuscript. YT, X-TT, and XL conceived the study, participated in the design, collected the samples, and helped to draft the manuscript. A-SW and H-SZ collected the samples. All authors have read and approved the final manuscript.

## Funding

Special application for the construction of innovative provinces in Hunan, China No. S2021SFYLJS0026.

## Conflict of Interest

The authors declare that the research was conducted in the absence of any commercial or financial relationships that could be construed as a potential conflict of interest.

## Publisher's Note

All claims expressed in this article are solely those of the authors and do not necessarily represent those of their affiliated organizations, or those of the publisher, the editors and the reviewers. Any product that may be evaluated in this article, or claim that may be made by its manufacturer, is not guaranteed or endorsed by the publisher.
