# Supplementary figures and images for: Comparison of Neoadjuvant Chemotherapy Efficiency in Advanced Ovarian Cancer Patients Treated With Paclitaxel Plus Carboplatin and Intraperitoneal Bevacizumab vs. Paclitaxel With Carboplatin
Source: Front Med (Lausanne). 2022 Mar 9;9:807377. doi: 10.3389/fmed.2022.807377 (PMC8959569; doi:10.3389/fmed.2022.807377)

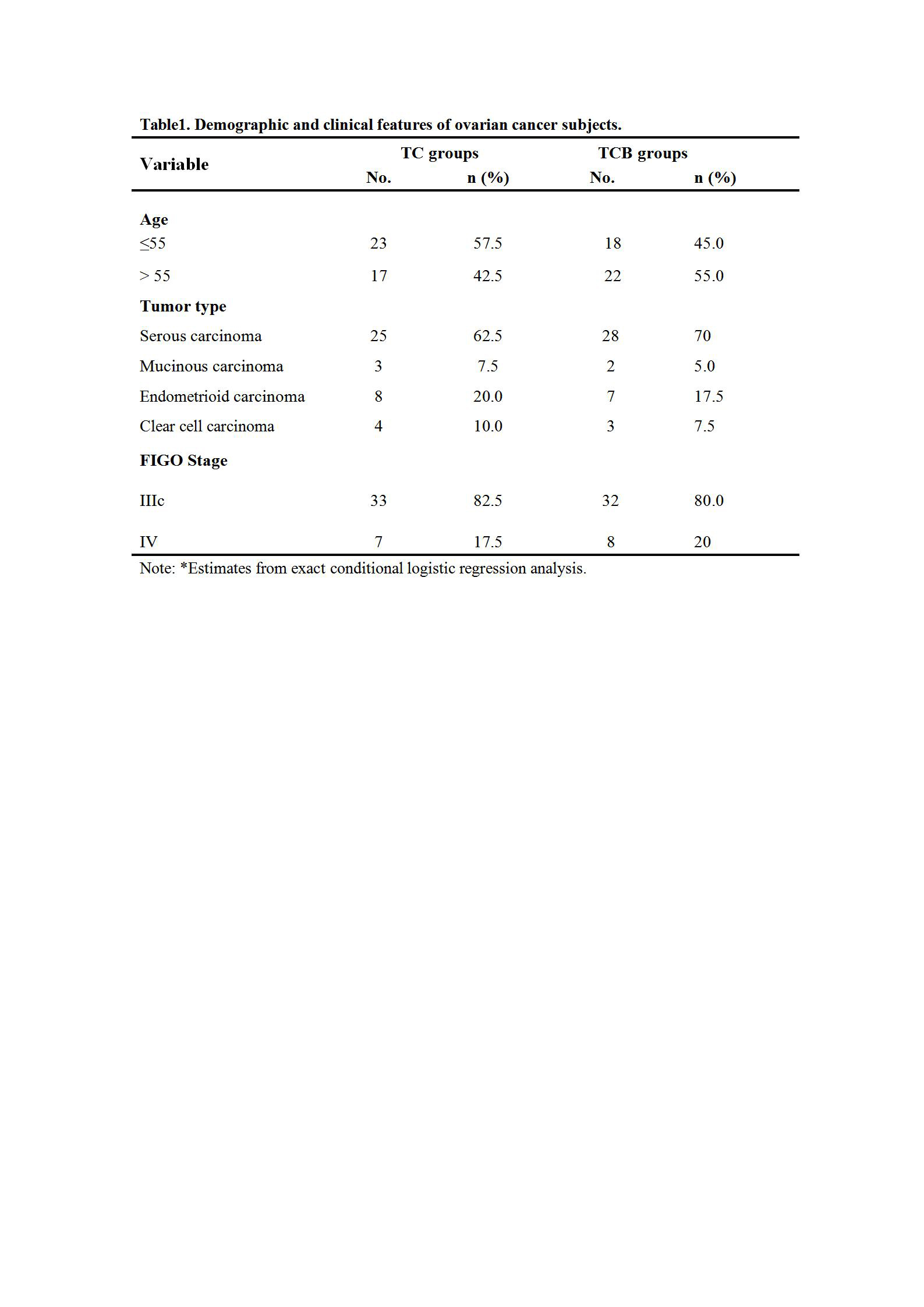

Supplement: Supplementary file 2 [file Image_1.JPEG]

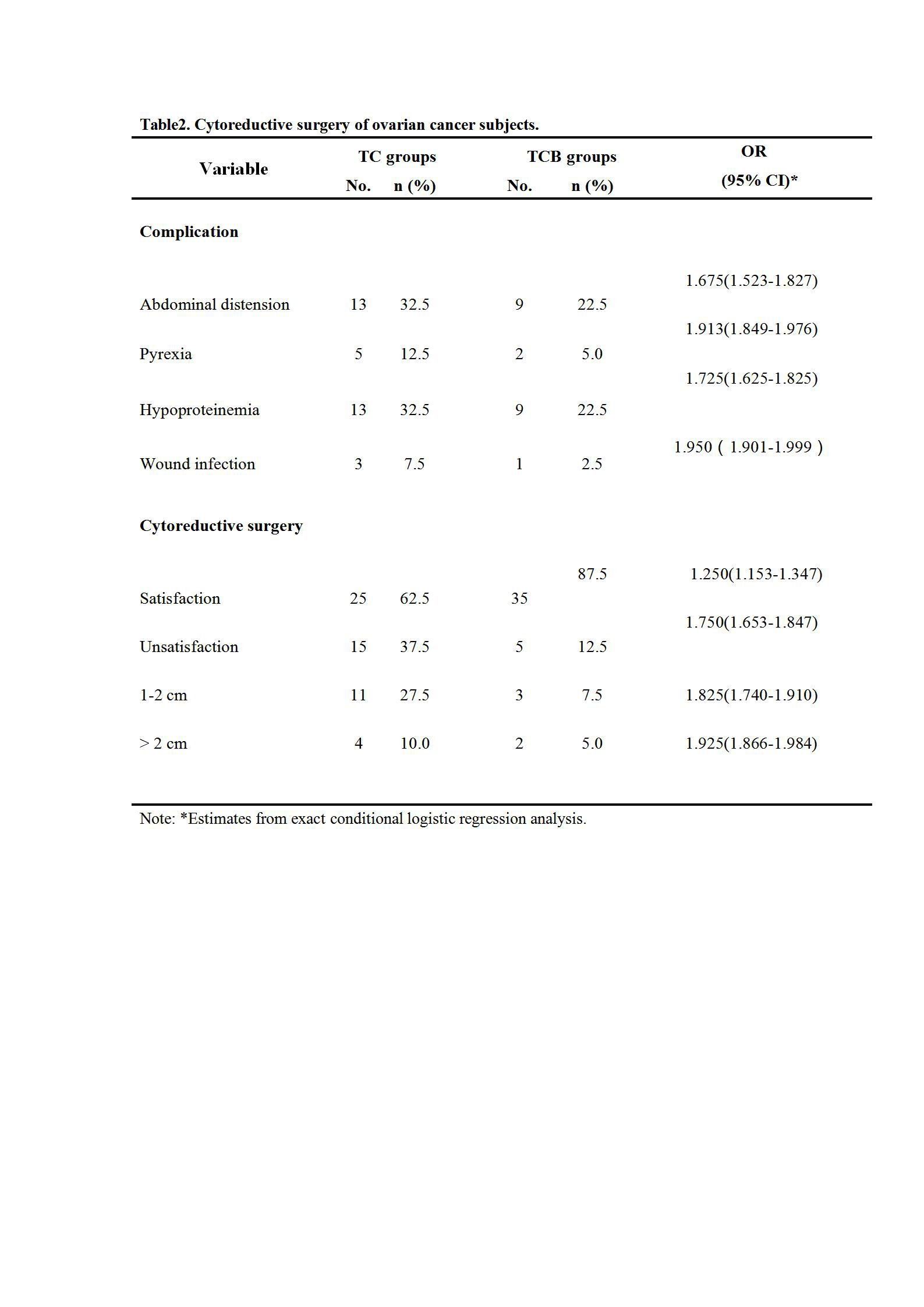

Supplement: Supplementary file 3 [file Image_2.JPEG]

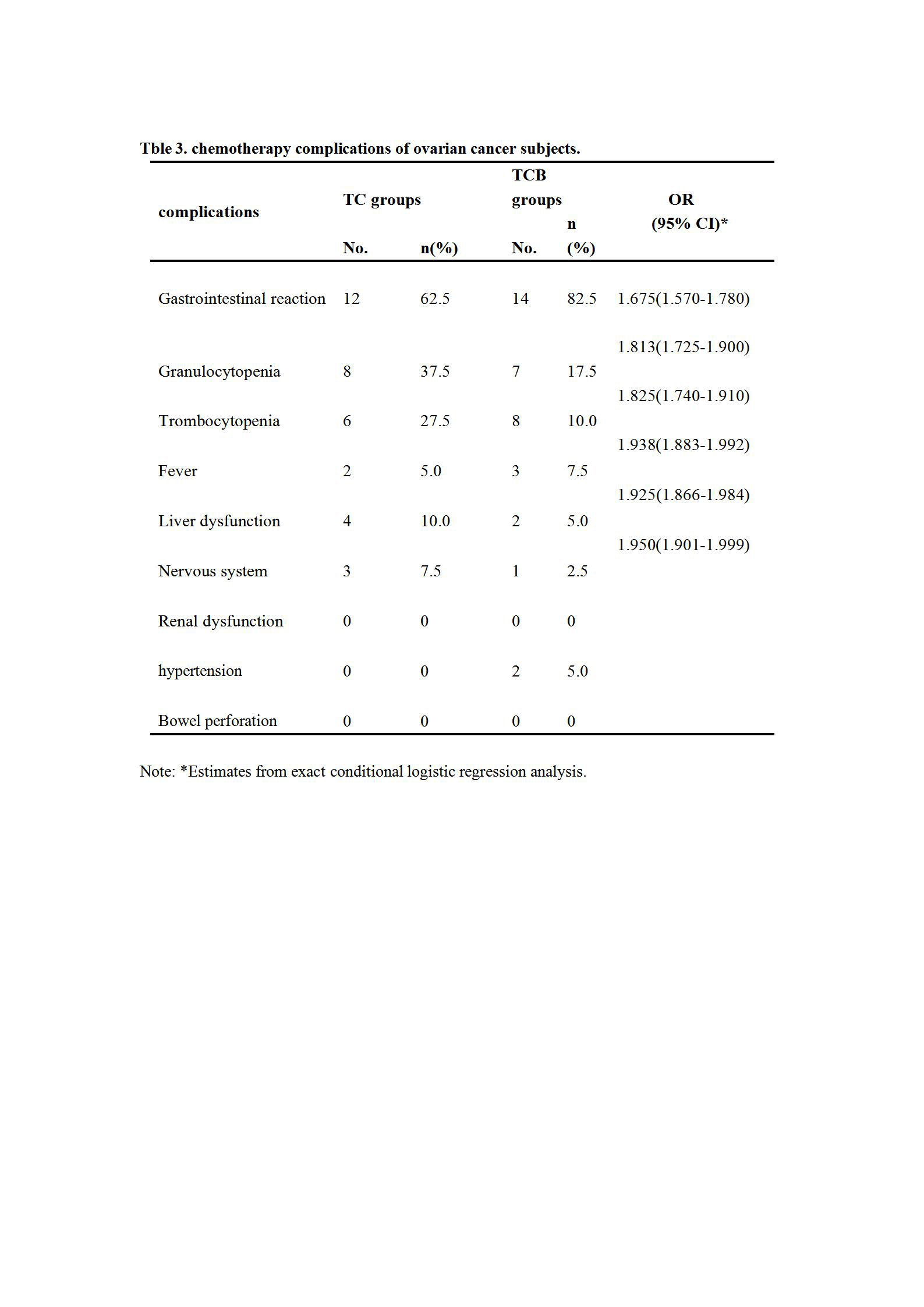

Supplement: Supplementary file 4 [file Image_3.JPEG]
